# Has collateral blood flow any effect on restenosis rate? Our experience

**DOI:** 10.3389/fneur.2024.1360161

**Published:** 2024-02-27

**Authors:** Yanjiang Li, Yujie Sun, Tonghui Liu, Peng Liu, Guangwen Li, Yong Zhang

**Affiliations:** Department of Neurology, The Affiliated Hospital of Qingdao University, Qingdao, Shandong, China

**Keywords:** restenosis, collateral circulation, intracranial, angioplasty, stroke

## Abstract

**Objectives:**

Restenosis is one of the important factors affecting the effectiveness of percutaneous transluminal angioplasty and stenting in the treatment of intracranial atherosclerotic stenosis. We aimed to clarify whether recruitable collateral flow could cause restenosis in patients treated with percutaneous transluminal angioplasty and stenting.

**Material and methods:**

Our study retrospectively analyzed patients with symptomatic severe intracranial atherosclerotic stenosis (≥70%) who underwent percutaneous transluminal angioplasty and stenting. We enrolled 28 patients with restenosis and 71 patients without restenosis. We analyzed baseline data, perioperative events, and follow-up results of patients in the two groups. Binary logistic regression analysis was used to identify restenosis predictors.

**Results:**

For preoperative stroke, the restenosis group had a greater likelihood of having a previous stroke (89.3%), which was less prevalent in the non-restenosis group (66.2%) (*P* = 0.020). The restenosis group had a higher rate of re-stroke (21.4 vs. 4.2%, *P* = 0.022). After binary logistic regression analysis, collateral circulation and residual stenosis were independent risk factors of restenosis, with overall risk (95% confidence intervals) of 5.034 (1.484–4.066, *P* < 0.001) and 1.064 (1.006–1.125, *P* = 0.030), respectively. Restenosis risk increased 1.456-fold for each collateral circulation grade increase. However, for each 1% increase in residual stenosis, restenosis risk increased by 5.9% (*P* = 0.03). The chance of restenosis is minimal when the residual stenosis rate after percutaneous transluminal angioplasty and stent implantation is 15.85%.

**Conclusions:**

Good collateral circulation was significantly associated with restenosis in patients undergoing intracranial angioplasty, the residual stenosis rate tends to be 15.85% to reduce restenosis risk. Compared to patients with restenosis, those without restenosis have a low stroke risk during follow-up.

## Introduction

Intracranial atherosclerotic stenosis (ICAS) is the most common cause of ischemic stroke (1). Despite optimal drug therapy, patients with ICAS experience a high rate of re-stroke, disability, and death (2). Percutaneous transluminal angioplasty and stenting (PTAS) is a potential treatment that restores anterograde blood flow by remodeling the blood vessels (3). However, the safety and efficacy of PTAS have been widely questioned because of the high incidence of perioperative complications and recurrent stroke. The SAMMPRIS and VISIT trials showed greater rates of stroke and death within 30 days in patients treated with PTAS (4, 5). In the CASSISS trial, percutaneous intervention was found to be an effective treatment for symptomatic ICAS after applying strict inclusion criteria (6). A *post-hoc* analysis of SAMMPRIS found that patients with new infarcts had a 66.7% rate of restenosis during long-term follow-up (7). Studies have identified many factors influencing restenosis after PTAS for symptomatic ICAS, including age, diabetes, lesion length, and residual stenosis rate after treatment (8–10). As an alternative blood supply route, the brain's collateral circulation may provide blood flow when blood vessels are blocked. Collaterals can effectively reduce the loss of ischemic penumbra and extend the time window for acute stroke treatment (11). However, a *post-hoc* analysis based on the original warfarin-aspirin symptomatic intracranial disease (WASID) trial population of 569 patients found that good collateral circulation likely leads to recurrent stroke in patients with mild stenosis (12).

To date, no study has determined the effect of collateral circulation on restenosis after intracranial PTAS. Therefore, this study was conducted to investigate the possible role of different grades of collateral circulation on the risk of restenosis after intracranial PTAS.

## Materials and methods

### Patient selection

We retrospectively analyzed the data of 99 patients treated with PTAS at the Affiliated Hospital of Qingdao University from 2019.1 to 2022.6. These patients met the following inclusion criteria: 1. symptomatic ICAS in patients undergoing PTAS, the symptoms include TIA or ischemic stroke; 2. the target vessels include the terminal internal carotid artery (C4–C7), the middle cerebral artery, the V4 segment of the vertebral artery and the basilar artery; 3. the rate of atherosclerotic stenosis was 70–99% as confirmed by DSA according to the WASID method (13); 4. willingness to be followed up, including undergoing a computed tomography angiography (CTA) or DSA; 5. more than 2 weeks from the onset of the ischemic event.

The exclusion criteria were: 1. another intracranial artery with stenosis >70%; patients with tandem lesions or stroke caused by perforator occlusion; 2. a non-atherosclerotic lesion, including cardioembolic stroke, moyamoya disease, vasculitis, dissection or tandem stenosis of extracranial and intracranial arteries; 3. concurrent intracranial pathology including tumors, aneurysms, or arteriovenous malformation (Table 1).

**Table 1:** Inclusion and exclusion criteria.

**Inclusion criteria**	
	1. Symptomatic ICAS in patients undergoing PTAS, the symptoms include TIA or ischemic stroke.
	2. The target vessels include the terminal internal carotid artery (C4–C7), the middle cerebral artery, the V4 segment of the vertebral artery and the basilar artery.
	3. The rate of atherosclerotic stenosis was 70–99% as confirmed by DSA according to the WASID method.
	4. Willingness to be followed up, including undergoing a computed tomography angiography (CTA) or DSA.
	5. More than 2 weeks from the onset of the ischemic event.
**Exclusion criteria**	
	1. Another intracranial artery with stenosis >70%; patients with tandem lesions or stroke caused by perforator occlusion.
	2. A non-atherosclerotic lesion, including cardioembolic stroke, moyamoya disease, vasculitis, dissection or tandem stenosis of extracranial and intracranial arteries.
	3. Concurrent intracranial pathology including tumors, aneurysms, or arteriovenous malformation.

This retrospective study was conducted in accordance with the Helsinki Declaration (as revised in 2013) ethical standards and was approved by the ethics committee of the Affiliated Hospital of Qingdao University.

### Perioperative management

All patients received dual antiplatelet therapy (aspirin 100 mg and clopidogrel 75 mg daily) for >5 days before the procedure. During the first 3 months after the intervention, the patient continued dual antiplatelet therapy. Subsequently, long-term administration of either aspirin (100 mg/day) or clopidogrel (75 mg/day) was applied to the patients. Statins were also administered for at least 6 months after stenting.

### Procedure

All patients were administered general anesthesia. Usually, a 6-F guiding catheter was used for device delivery. A balloon (Boston Scientific, Natick, MA, USA) was placed in the stenotic segment for submaximal angioplasty. For initial treatment, primary balloon angioplasty alone is performed; stenting is considered in patients with insufficient dilation, marked acute dissection, or restenosis. The operators were instructed to choose whether to use stenting based on the morphological features of the lesion. The stent was placed at the lesion site after a submaximal angioplasty. After 15 min of observation, cerebral arteriography was repeated. The procedure was completed if the stent position was satisfactory and no residual stenosis >50% or acute thrombosis was observed.

Non-contrast head computed tomography (CT) was performed to exclude hemorrhage after the procedure. If patients were free of hemorrhage, GP IIb/IIIa receptor inhibitors were administered to those with a high risk of thrombosis. Blood pressure was maintained below 130/80 mmHg to prevent hyperperfusion syndrome.

### Data collection and follow-up

**Figure 1:**
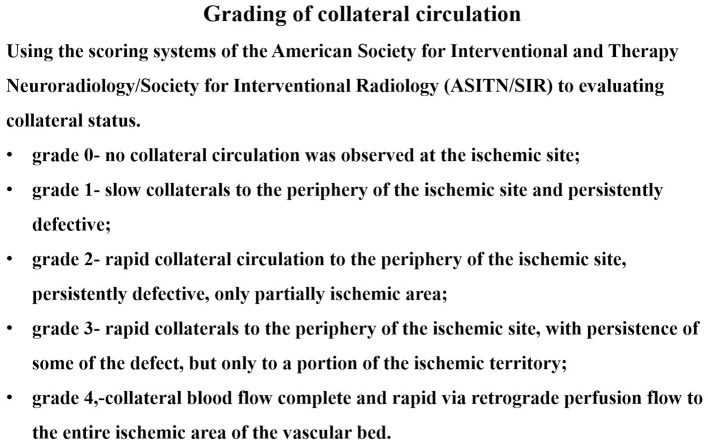
Grading of collateral circulation.

Baseline demographics; vascular risk factors; and clinical, angiographic, and periprocedural data were collected. Vascular imaging studies, including CTA and DSA, were scheduled 12 months after the index procedure. Two experienced neurointerventionists interpreted the images. Collateral circulation was graded using the Astin/Sir Score (Figure 1). The primary endpoints were periprocedural complications (death, ischemic stroke, or hemorrhagic stroke), transient ischemic attacks, ischemic stroke, restenosis, and symptomatic restenosis. Symptomatic restenosis was defined as restenosis associated with TIA or stroke in the offending vessel territory. The WASID technique was used to calculate the percentage of patients with residual or recurrent stenosis. For patients evaluated using cerebral angiography, restenosis was defined as having lesions with >50% stenosis. The lesion locations were considered restenosis for patients evaluated using CTA if both the treated segment and the edge of the treated segment could not be displayed well or showed an apparent filling defect on CTA.

### Statistical analysis

Statistical analysis was performed using SPSS version 26.0 for Windows (Armonk, NY: IBM Corp; 2019). Normality was assessed for continuous variables using the Shapiro–Wilk test. Continuous variables are expressed as the mean ± standard deviation (SD) or as the median with interquartile range and compared using Student's *t*-test or the Mann–Whitney *U*-test, as appropriate. Categorical variables were expressed as rates and compared with the chi-squared test or Fisher exact test; α = 0.05 was used as the test standard. Univariate analysis and the binary logistic regression model were performed on the data. The optimal residual stenosis rate was expressed using the receiver operating characteristic (ROC) curve.

## Results

Ninety-nine patients were enrolled, including 27 treated with balloon angioplasty and 72 treated with balloon dilatation plus stenting. We enrolled 28 patients with restenosis and 71 patients without restenosis. Hypertension was the most common risk factor in both groups (*n* = 4, 74.7%). Anterior circulation lesions were present in 53.5% of patients; no differences were found in age, sex, proportion of risk factors, and lesion location (*P* > 0.05). For preoperative stroke, the restenosis group had a greater likelihood of having a previous stroke (89.3%), which was less prevalent in the non-restenosis group (66.2%; *P* = 0.020) (Table 2).

**Table 2:** The baseline characteristics of the restenosis and the non-restenosis groups.

**Variable**	**All (*n* = 99)**	**Restenosis (*n* = 28)**	**Non-restenosis (*n* = 71)**	***P*-value**
Age, mean ± SD	61.74 ± 8.34	62.64 ± 7.32	59.54 ± 9.16	0.147
BMI, mean ± SD	25.94 ± 3.04	25.6 ± 3.50	26.08 ± 2.85	0.529
Male, *n* (%)	67 (67.7%)	16 (57.1%)	51 (71.8%)	0.159
Hypertension, *n* (%)	74 (74.7%)	23 (82.1%)	51 (71.8%)	0.288
Smoking, *n* (%)	32 (32.3%)	5 (17.9%)	27 (38.0%)	0.053
Diabetes mellitus, *n* (%)	34 (34.3%)	10 (35.7%)	24 (33.8%)	0.857
Hyperlipidemia, *n* (%)	25 (25.3%)	6 (21.4%)	19 (27.1%)	0.588
**Qualifying ischemic events**				
Ischemic stroke	72 (72.7%)	25 (89.3%)	47 (66.2%)	0.020
TIA	27 (27.3%)	3 (10.7%)	24 (33.8%)	
**Lesion distribution**				
Anterior circulation	53 (53.5%)	18 (64.3%)	35 (49.3%)	0.178
Posterior circulation	46 (46.5%)	10 (35.7%)	36 (50.7%)	

During the operation, all stents were successfully delivered to the lesion; none failed to reach the location. The differences in the length and diameter of the used balloons and stents were not statistically significant between the two groups. Stenosis degrees after intervention [18.65 (10.17) vs. 14.30 (13.60), *P* = 0.04)] were significantly lower in the non-restenosis group than that in the restenosis group (Table 3).

**Table 3:** Periprocedural and technical characteristics.

**Variable**	**Restenosis (*n* = 28)**	**Non-restenosis (*n* = 71)**	***P*-value**
Stenosis rate preoperatively, (Q25)	90 (13.75)	90 (10)	0.047
Residual stenosis rate, (Q25)	18.65 (10.17)	14.30 (13.60)	0.04
Diameter of the balloon	2 (0.94)	2 (0.5)	0.266
Length of balloon	15 (0.00)	13.5 (5.00)	0.204
Diameter of the stent	3.25 (2.00)	3 (2.00)	0.876
Length of stent	16.5 (9.00)	13 (7.00)	0.451
Astin/Sir			< 0.01
0	1	18	
1	5	28	
2	4	12	
3	15	13	
4	3	1	

In the restenosis and non-restenosis groups, the rates of any cause of safety endpoints within 30 days showed no statistical difference. In the restenosis group, one patient developed an asymptomatic intracerebral hemorrhage after surgery; the occurrence of restenosis was associated with stroke during follow-up [21.4% of restenosis patients (*n* = 6) vs. 4.2% of non-restenosis patients (*n* = 3); *P* = 0.022]. No significant differences were found in periprocedural complications, cerebral hemorrhage, death, and other characteristics (Table 4).

**Table 4:** Clinical and radiological outcomes.

	**Restenosis (*n* = 28)**	**(*n* = 71)**	***P*-value**
30-day ischemic stroke	0	0	1
30-day hemorrhagic stroke	1 (3.6%)	0	0.628
30-day death	0	0	1
30 days to 1-year ischemic stroke	6 (21.4)	3 (4.2)	0.022
30 days to 1-year territory ischemic stroke	5 (17.9)	2 (2.8)	0.028
30 days to 1-year hemorrhagic stroke	0	0	1
30 days to 1-year death	0	0	1

**Figure 2:**
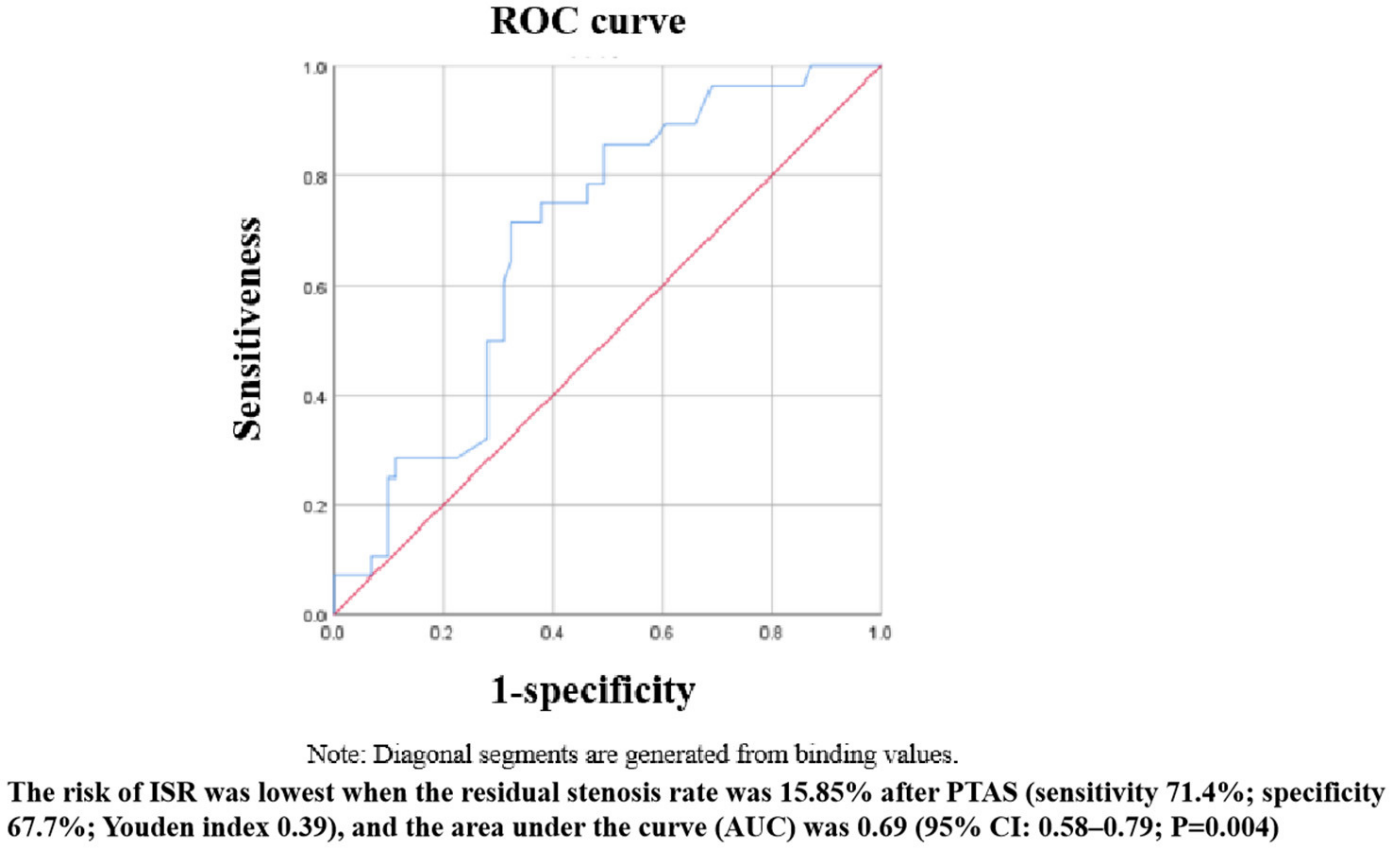
Receiver operating characteristic curve.

After binary logistic regression, we found that the Astin/Sir [odds ratio (OR) 5.034, 95% confidence interval (CI) 1.484–4.066; *P* < 0.001] and more severe residual stenosis (OR 1.064, 95% CI 1.006–1.125; *P* = 0.030) were independently associated with restenosis (Table 5). We constructed a binary logistic regression prediction model for restenosis after ICAS in the form of a nomogram, which was used to predict the effect of residual stenosis on restenosis. The risk of ISR was lowest when the residual stenosis rate was 15.85% after PTAS (sensitivity 71.4%; specificity 67.7%; Youden index 0.39), and the area under the curve (AUC) was 0.69 (95% CI: 0.58–0.79; *P* = 0.004) (Figure 2).

**Table 5:** Analysis of risk factors for restenosis after PTAS according to binary logistic regression analysis.

	**B-value**	**OR value (95% CI)**	***P*-value**
Astin/sir	0.899	4.066 (1.484–2.456)	< 0.001
Stenosis rate preoperatively	0.057	1.148 (0.977–1.059)	0.161
Residual stenosis rate	0.062	1.125 (1.006–1.064)	0.03
Smoking	1.053	9.802 (0.838–2.866)	0.093

## Discussion

This study identified that poor collateral circulation could reduce the incidence of restenosis after PTAS for symptomatic ICAS. Compared to the no-restenosis group, the restenosis group had an increased risk of stroke recurrence during follow-up. Our analysis showed that reducing residual stenosis within a certain range could lower the restenosis rate.

A high restenosis rate is a frustrating outcome of PTAS (14). In a *post-hoc* SAMMPRIS analysis, 66.7% of cases with an ischemic event beyond 30 days were attributed to restenosis of the offending vessel during the follow-up. In patients with Wingspan stent implantation, the symptomatic restenosis rates at 1, 2, and 3 years were 9.6, 11.3, and 14%, respectively (7). In our study, 66.7% of the re-stroke patients had restenosis. Among patients with ipsilateral ischemic stroke, this rate was 71.4% (5/7). The specific influencing factors of restenosis remain inconclusive. Collateral circulation may be an important reason for this phenomenon.

Collateral circulation is closely related to stroke in patients with ICAS and has a protective effect on severe stenosis. People with good collateral circulation can tolerate ischemia longer and have a smaller core infarct volume than those with poor collateral circulation. The rate of progression from penumbra to infarction depends largely on the extent of collateral circulation and ultimately determines whether the patient has a fast or slow progression. Previous studies have shown that collateral circulation plays an important role in the occurrence of restenosis and strongly promotes the development of re-stroke (15, 16). Other studies found that collateral circulation might not predict the risk of restenosis (17). This phenomenon may be attributed to the differences in measurement and evaluation methods of collateral circulation.

In our study, people with good collateral circulation were more likely to develop restenosis after PTAS. The cause for this phenomenon may include the recruitment of collateral vessels. Collateral arteries in healthy tissues have little or no net collateral flow. In ischemic tissue, the pressure gradient exerted upon the collateral vasculature increases the blood flow of collateral circulation. Blood flow along collateral circulation comes from opposite directions. This “to and fro” flow increases when the vessel stenosis is relieved (18). This competitive flow may lower shear stress through these vessels dramatically (19). Local hemodynamic factors, low shear stress in particular, are known to critically affect the natural history of atherosclerosis. Increasing evidence now suggests that low shear stress may contribute to the development of restenosis (20). The proliferation of smooth muscle cells is one of the main reasons that promote the development of restenosis. Low shear stress promotes restenosis through interactions of shear sensing endothelial cells with smooth muscle cells (21, 22). Abundant collateral circulation may exacerbate this phenomenon. In addition, the proliferation of blood vessels significantly influences collateral circulation. A considerable amount of variability is evident in collateral circulation. The ideal configuration of the collaterals is reported in only a minority of cases. Mostly, the body requires arteriogenesis to relieve tissue ischemia. Unlike healthy collateral arteries, arterial neogenesis leads to significant tortuosity of collaterals, remodeling of the luminal diameter, and hemodynamic changes (23). This may further increase the risk of restenosis. Previous studies have found that the mechanisms of restenosis include smooth muscle cell proliferation, thrombus formation, intimal hyperplasia, and atherogenic plate formation (24). Changes in the flow shear stress and pressure gradient indicate a greater possibility of thrombus formation and lipid accumulation.

Submaximal angioplasty is an effective tool for the treatment of patients with ICAS. By marginally increasing the diameter of the affected blood vessel, blood flow is markedly increased, thus alleviating the patient's hypoperfusion-type symptoms. The specific degree of dilation of intracranial blood vessels has not yet been determined. Underexpansion appears to be an important mechanism of restenosis. However, excessive deployment of the balloon and stent can reduce the vessel curvature and change the hemodynamic pattern, inevitably leading to the destruction of endothelial cells and triggering neuroinflammation, ultimately causing restenosis (25, 26). Excessive dilatation also may lead to intimal damage and increase the risk of intracerebral hemorrhage. A *post-hoc* analysis revealed that the sensitivity and specificity curves identified different thresholds for the optimal stent expansion area after the intervention (27). Therefore, an appropriately sized balloon should be chosen to balance the risk of restenosis and perioperative complications. We found that the risk of ISR was lowest when the residual stenosis rate was 15.85% after PTAS. The sensitivity of the ROC curve was a little low, which may be related to the small sample size in the ISR group.

## Limitations

First, this study was a retrospective analysis. We enrolled patients who were willing to undergo follow-up imaging. Potential biases included patient selection and quantification of the degree of stenosis. Second, the small sample size collected in this study may cause statistical bias. In addition, because of the controversy regarding the conclusion of the PTAS end, we selected only patients who still had symptomatic ICAS despite receiving medical therapy.

## Data availability statement

The original contributions presented in the study are included in the article/supplementary material, further inquiries can be directed to the corresponding authors.

## Ethics statement

The studies involving humans were approved by the Ethics Committee of the Affiliated Hospital of Qingdao University (QYFY WZLL 28202). The studies were conducted in accordance with the local legislation and institutional requirements. Written informed consent for participation was not required from the participants or the participants' legal guardians/next of kin in accordance with the national legislation and the institutional requirements. The study was conducted in accordance with the 1964 Helsinki Declaration and its later amendments or comparable ethical standards.

## Author contributions

YL: Conceptualization, Writing – original draft, Writing – review & editing. YS: Data curation, Methodology, Writing – review & editing. TL: Supervision, Validation, Writing – review & editing. PL: Conceptualization, Investigation, Writing – review & editing. GL: Data curation, Methodology, Writing – review & editing. YZ: Resources, Validation, Writing – review & editing.
